# Stereotactic body radiotherapy for central non-small cell lung cancer: risk analysis of radiation pneumonitis and bronchial dose constraints

**DOI:** 10.1093/jrr/rraf016

**Published:** 2025-04-14

**Authors:** Nozomi Kita, Natsuo Tomita, Taiki Takaoka, Machiko Ukai, Dai Okazaki, Masanari Niwa, Akira Torii, Seiya Takano, Masanosuke Oguri, Akane Matsuura, Yuto Kitagawa, Yuta Eguchi, Akio Niimi, Akio Hiwatashi

**Affiliations:** Department of Radiology, Nagoya City University Graduate School of Medical Sciences, 1 Kawasumi, Mizuho-cho, Mizuho-ku, Nagoya, Aichi 467-8601, Japan; Department of Radiology, Nagoya City University Graduate School of Medical Sciences, 1 Kawasumi, Mizuho-cho, Mizuho-ku, Nagoya, Aichi 467-8601, Japan; Department of Radiology, Nagoya City University Graduate School of Medical Sciences, 1 Kawasumi, Mizuho-cho, Mizuho-ku, Nagoya, Aichi 467-8601, Japan; Department of Radiology, Nagoya City University Graduate School of Medical Sciences, 1 Kawasumi, Mizuho-cho, Mizuho-ku, Nagoya, Aichi 467-8601, Japan; Department of Radiology, Nagoya City University Graduate School of Medical Sciences, 1 Kawasumi, Mizuho-cho, Mizuho-ku, Nagoya, Aichi 467-8601, Japan; Department of Radiology, Nagoya City University Graduate School of Medical Sciences, 1 Kawasumi, Mizuho-cho, Mizuho-ku, Nagoya, Aichi 467-8601, Japan; Department of Radiology, Nagoya City University Graduate School of Medical Sciences, 1 Kawasumi, Mizuho-cho, Mizuho-ku, Nagoya, Aichi 467-8601, Japan; Department of Radiology, Nagoya City University Graduate School of Medical Sciences, 1 Kawasumi, Mizuho-cho, Mizuho-ku, Nagoya, Aichi 467-8601, Japan; Department of Radiology, Nagoya City University Graduate School of Medical Sciences, 1 Kawasumi, Mizuho-cho, Mizuho-ku, Nagoya, Aichi 467-8601, Japan; Department of Radiology, Nagoya City University Graduate School of Medical Sciences, 1 Kawasumi, Mizuho-cho, Mizuho-ku, Nagoya, Aichi 467-8601, Japan; Department of Radiology, Nagoya City University Graduate School of Medical Sciences, 1 Kawasumi, Mizuho-cho, Mizuho-ku, Nagoya, Aichi 467-8601, Japan; Department of Radiology, Nagoya City University Graduate School of Medical Sciences, 1 Kawasumi, Mizuho-cho, Mizuho-ku, Nagoya, Aichi 467-8601, Japan; Department of Respiratory Medicine, Allergy and Clinical Immunology, Nagoya City University Graduate School of Medical Sciences, 1 Kawasumi, Mizuho-cho, Mizuho-ku, Nagoya, Aichi 467-8601, Japan; Department of Radiology, Nagoya City University Graduate School of Medical Sciences, 1 Kawasumi, Mizuho-cho, Mizuho-ku, Nagoya, Aichi 467-8601, Japan

**Keywords:** stereotactic body radiotherapy, non-small cell lung cancer, radiation pneumonitis, toxicity

## Abstract

The present study investigated risk factors and bronchial dose constraints for symptomatic radiation pneumonitis (RP) in stereotactic body radiotherapy (SBRT) for central early-stage non-small cell lung cancer (NSCLC). We reviewed 245 patients with early-stage NSCLC treated with SBRT, and 78 patients with a tumor within 3 cm of the main or lobar bronchus were included in this study. Dose-volume histogram data were converted to a 4-fraction equivalent using the linear-quadratic model with an α/β value of 3. To examine the independent effects of dose parameters on grade ≥ 2 RP after adjusting for clinical factors, the Fine-Gray model with death as a competing risk was used for evaluation. With a median follow-up period of 44 months, the 4-year cumulative incidence of grade ≥ 2 and ≥ 3 RP was 22.5% and 8.5%, respectively. After adjustment for clinical factors, 6 bronchial dosimetric factors were significantly associated with grade ≥ 2 RP. Lung dosimetric factors were not significantly associated with grade ≥ 2 RP. Among significant dosimetric factors of the bronchus, bronchus V35_Gy_ had the highest hazard ratio (HR) (HR 1.24, 95% CI 1.03–1.49, *P* = 0.027). The optimal threshold for bronchus V35_Gy_ based on receiver operating characteristic curve analysis was 0.04 cc. The 4-year incidence of grade ≥ 2 RP in the bronchus V35_Gy_ ≤ 0.04 cc vs. >0.04 cc groups was 15.7% vs. 37.0% (*P* = 0.036). In SBRT for central early-stage NSCLC, bronchus V35_Gy_ < 0.04 cc is the definitive indicator for preventing grade ≥ 2 RP.

## INTRODUCTION

Recent studies suggested that stereotactic body radiotherapy (SBRT) is a viable treatment option not only for medically inoperable patients with early-stage non-small cell lung cancer (NSCLC), but also for operable cases [1–3] and oligometastatic cancers [4–7]. SBRT is generally a safe and highly effective treatment for lung tumors and severe toxicity is less likely to occur when SBRT is employed for peripheral tumors [8–10]. On the other hand, the central tumor, which was originally defined as being located within 2 cm of the main or lobar bronchus [11, 12], is an independent risk factor for severe toxicity, such as radiation pneumonitis (RP) and hemorrhage, after SBRT [11, 13, 14]. Since severe toxicities were reported following the delivery of SBRT at doses of 60–66 Gy in three fractions to central tumors, a risk-adapted dosing strategy may be employed for central tumors [8]. However, even when dose fractionation regimens with reduced biologically effective doses (BED) were attempted, severe toxicities, including treatment-related death, occurred [15–17].

In our previous study, we reported that a central tumor was an independent risk factor for RP in SBRT for early-stage NSCLC [18]. The reasons for central tumors being a risk factor may include inflammation of the bronchus, lymphatic flow and an increased radiation dose to the lungs; however, the exact cause remains unclear [19, 20]. We specifically hypothesized that the dose to the bronchus might be associated with the development of RP. Based on these findings, when SBRT is employed for central tumors, it is important to clarify whether the delivery of doses to the lung or bronchus will contribute more to the development of RP. In addition, if the relationship between the dose to the bronchus and the development of RP is proven, bronchial dose constraints may be helpful for evaluating the risks of toxicities in SBRT plans for central tumors. Therefore, the present study investigated risk factors and bronchial dose constraints for symptomatic RP in SBRT for central early-stage NSCLC.

## MATERIALS AND METHODS

### Study population

**Fig. 1:**
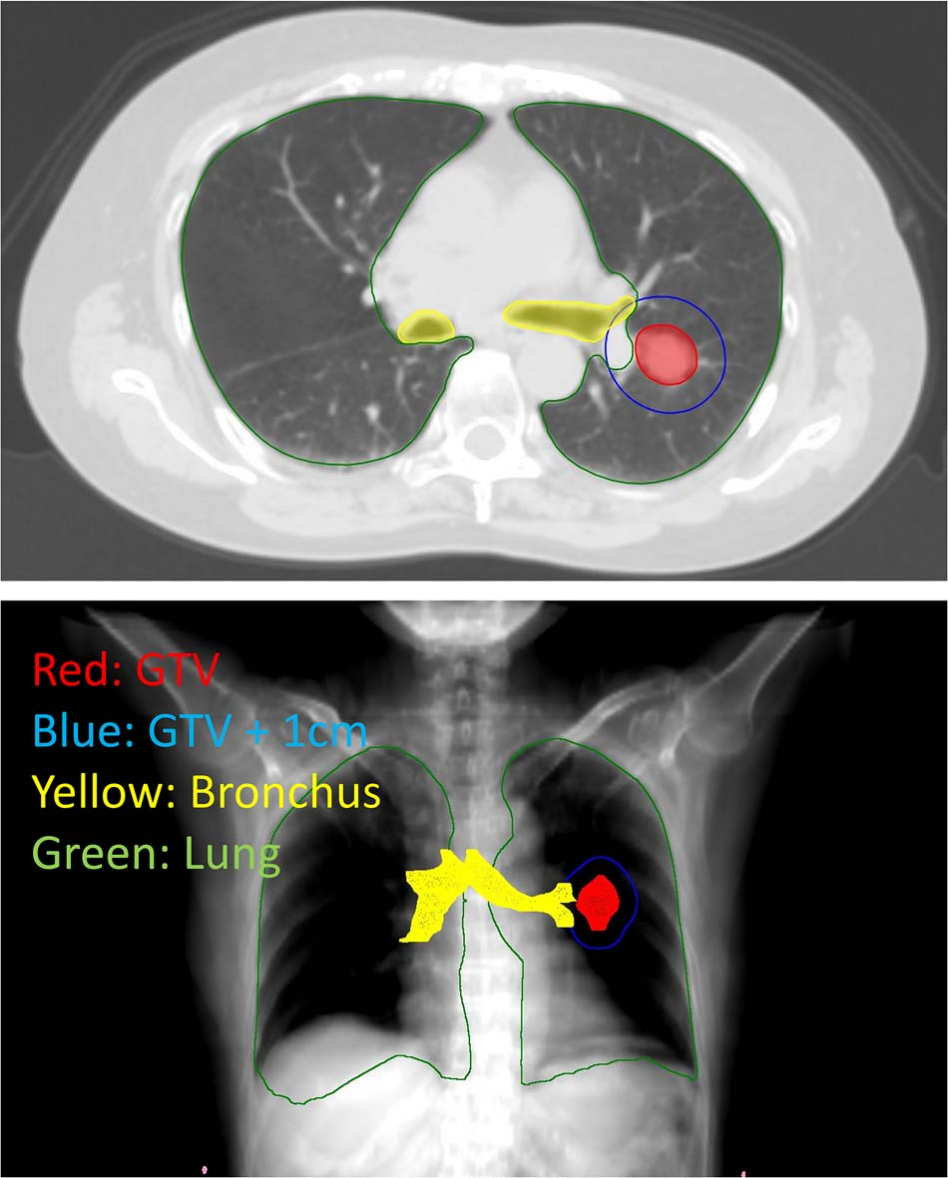
Example of the delineation of a tumor within 1 cm of the main or lobar bronchus. Illustration of distances from the tumor and its relative position to the bronchus: GTV, GTV + 1 cm and bronchus. The line representing GTV + 1 cm overlaps with the bronchus.

In the present study, patients with early-stage NSCLC treated with SBRT at our institution were analyzed. Inclusion criteria were as follows: (1) treatment with SBRT between February 2004 and September 2018; (2) Tis-T2bN0M0 clinical stage according to the 8th TNM classification; (3) NSCLC confirmed through a histological examination or strongly suspected based on diagnostic imaging and clinical progression; (4) tumors located within 3 cm of the main or lobar bronchus. Patients without available dose-volume histogram (DVH) data and those with a follow-up period of less than 3 months were excluded from the analysis. In the present study, the definition of the central tumor was expanded from the original definition [11] because not only high-dose parameters, including the maximum dose and D1cc, but also moderate-dose parameters, such as V20–30_Gy_, needed to be evaluated. Figure 1 shows an example of a selected patient with tumors located within 1 cm of lobar bronchus. This study was approved by our Institutional Review Board (approval number: 60-22-0024). Due to the retrospective nature of this study, the requirement for written informed consent was waived, and the study details were made publicly available through an opt-out notice on the website. This study was conducted in accordance with the Declaration of Helsinki and its later amendments.

### Pretreatment evaluation and SBRT methods

Details of the SBRT method were reported in our previous studies [18, 21, 22]. Eligibility for SBRT was confirmed based on evaluations by a multispecialty oncology committee. A 6 MV photon beam was used for SBRT, with the dose prescribed to the isocenter of the planning target volume (PTV). The PTV was created by adding a 5–10 mm margin in the cranio-caudal direction and a 5 mm margin in other directions to the internal target volume. Doses of 44–52 Gy in four fractions were prescribed based on the respective tumor diameters [18, 21, 22]. SBRT was administered twice a week in four fractions based on our radiobiological considerations [23, 24]. Regarding cases located in proximity to the pulmonary hilum or vital organs, individualized doses, such as 60 Gy in eight fractions, were administered at the discretion of the attending radiation oncologist. Patients were treated with the following prescribed doses: 44 Gy in four fractions (*n* = 1), 48 Gy in four fractions (*n* = 23), 50 Gy in four fractions (*n* = 20), 52 Gy in four fractions (*n* = 26), 54 Gy in six fractions (*n* = 1), 56 Gy in eight fractions (*n* = 1) and 60 Gy in eight fractions (*n* = 6).

### Dose-volume data and dose correction

Data on DVH and dose distributions were evaluated using RayStation (RaySearch Medical Laboratories AB, Stockholm, Sweden). A radiation oncologist re-contoured the bronchus, including the main and lobar bronchi, and evaluated DVH data. Figure 1 shows an example of the delineation of the bronchus and the distance between GTV and the bronchus. Regarding the bronchus, the mean bronchial dose and doses of Dmax, D0.1–0.5_cc_, D1–5_cc_ and V10–50_Gy_ were extracted from DVH. The mean lung dose (MLD) and V5–20_Gy_ were extracted from DVH for the lung as described in a previous study that examined the risk factors for RP in SBRT [18]. Among patients treated with 6 or 8 fractions, DVH data were converted to a 4-fraction equivalent using the linear-quadratic (LQ) model with an α/β value of 3.

### Follow-up evaluation and statistical analysis

Follow-up CT were done every 2 to 3 months for the first 6 months and then every 6 months thereafter. Additional tests such as blood tests, MRI or brain CT, and FDG-PET were performed as needed. The endpoint was established as the development of grade ≥ 2 RP. RP was diagnosed using clinical observations, blood tests, chest X-rays and CT. The grades of RP and other toxicities were defined in accordance with the CTCAE v5.0 criteria.

In the univariate analysis, the Gray test was used to evaluate the association between risk factors and grade ≥ 2 RP with death as a competing risk. Univariate analysis was performed using the following risk factors: age, sex, performance status, smoking history, interstitial pneumonia, forced expiratory volume in 1 second, tumor location, tumor diameter, distance from the bronchus and BED_10_. BED_10_ was used as a dosimetric factor. To examine the independent effects of dosimetric factors on grade ≥ 2 RP after adjusting for clinical factors, the Fine-Gray model with death as a competing risk was used for evaluation. Clinical factors with *P*-value <0.05 in univariate analysis and each dosimetric factor were included in the multivariate model. In the multivariate model, dosimetric factors were analyzed as continuous variables. The optimal threshold for the dosimetric factor was determined using a receiver operating characteristic (ROC) curve. Statistical analyses were performed using EZR, a graphical user interface for R (The R Foundation for Statistical Computing, Vienna, Austria) [25].

## RESULTS

A total of 245 patients with early-stage NSCLC were treated with SBRT at our institution between February 2004 and September 2018. Eighty patients had tumors located within 3 cm of the main or lobar bronchus. One patient was excluded due to unavailability of DVH data, and another was excluded due to a follow-up period of less than 3 months, resulting in 78 patients were analyzed. Patient characteristics are shown in Table 1. Tumors were located within 1 cm of the bronchus in 23 cases, between 1 and 2 cm in 19 cases and between 2 and 3 cm in 36 cases, with a median of 1.9 cm (range, 0–3.0). Supplementary Table 1 shows mean values of bronchial parameters, such as mean and maximum doses and D1_cc_, according to each clinical factor. Among the 78 patients analyzed, the maximum dose and bronchus D1_cc_ were significantly higher in lower lobe cases than in upper or middle lobe cases (*P* = 0.024 and 0.025). The mean and maximum doses and bronchus D1_cc_ were significantly higher in patients with tumors >26 mm than in those with tumors ≤26 mm (*P* = 0.01, 0.01 and 0.014).

**Table 1:** Patient and treatment characteristics

**Characteristics**	**Number or median**	**% or range**
Age (years)	77	58–88
Sex		
Male	43	55%
Female	35	45%
PS		
0	35	45%
1	36	46%
2	7	9%
Smoker		
Current	21	27%
Ex	30	38%
Non	27	35%
Interstitial pneumonia		
Yes	2	3%
No	76	97%
FEV_1_ (L)	1.49	0.65–2.64
Tumor location		
Upper or middle lobe	50	64%
Lower lobe	28	36%
Tumor diameter (mm)	26	0–50
Distance from the bronchus (cm)	1.9	0.0–3.0
≤1	23	29%
1–2	19	24%
2–3	36	46%
Total dose	50	44–60
Fractions	4	4–8
BED_10_	112.5	92.4–119.6

PS, performance status; FEV_1_, forced expiratory volume in 1 second; BED_10_, biologically effective dose calculated with an α/β value of 10.

The median follow-up period was 44 months (range, 3–198) for all patients and 60 months (range, 4–198) for living patients. Grades 1, 2 and 3 RP developed in 50 (64.1%), 11 (14.1%) and six patients (7.7%), respectively. No cases of grade ≥ 4 RP were observed. Other grade ≥ 2 adverse events included the following: pleural effusion in three cases, cough in two, dyspnea in three, hemoptysis in one, rib fracture in six, chest wall pain in four and dermatitis in one. Among the 78 patients analyzed, 17 (21.8%) and six patients (7.7%) developed grade ≥ 2 and ≥ 3 RP, respectively. The median time for the onset of grade ≥ 2 and ≥ 3 RP was 5 (range, 2–13) and 5.5 (range, 3–36) months after SBRT, respectively. The 4-year cumulative incidence of grade ≥ 2 and ≥ 3 RP was 22.5% (95% confidence interval [CI], 13.8–32.6) and 8.5% (95% CI, 3.4–16.5), respectively.

Gray’s test was used to examine the relationships between risk factors and grade ≥ 2 RP. Table 2 shows differences in the incidence of grade ≥ 2 RP according to each risk factor. Age, tumor diameter, distance from the bronchus and BED_10_ correlated with the development of grade ≥ 2 RP (*P* = 0.002, <0.001, 0.021 and 0.019, respectively).

**Table 2:** Cumulative incidence of grade ≥ 2 radiation pneumonitis according to each risk factor

**Characteristics**	**Number**	**4-year incidence**	**95% CI**	***P*-value**
Age (years)				0.002
≤77	41	7.9%	2.0–19.4	
>77	37	37.8%	22.4–53.2	
Sex				0.14
Male	43	29.0%	16.1–43.4	
Female	35	14.8%	5.3–28.8	
PS				0.76
0, 1	71	21.9%	12.9–32.3	
2	7	NA	NA	
Smoker				0.073
Yes	51	28.8%	16.8–41.9	
No	27	11.3%	2.7–26.5	
Interstitial pneumonia				0.44
Yes	2	NA	NA	
No	76	21.8%	13.1–31.9	
FEV_1_ (L)				0.74
≤1.49	39	23.4%	11.4–37.8	
>1.49	39	21.6%	10.0–36.1	
Tumor location				0.24
Upper or middle lobe	50	18.7%	9.2–31.0	
Lower lobe	28	29.2%	13.6–46.9	
Tumor diameter (mm)				<0.001
≤26	41	5.3%	0.9–15.7	
>26	37	40.8%	24.8–56.2	
Distance from the bronchus (cm)				0.021
≤1.9	41	33.0%	18.9–47.9	
>1.9	37	11.2%	3.5–24.1	
BED_10_				0.019
≤112.5	52	14.1%	6.1–25.3	
>112.5	26	38.7%	20.1–57.1	

95% CI, 95% confidence interval; PS, performance status; FEV_1_, forced expiratory volume in 1 second; BED_10_, biologically effective dose calculated with an α/β value of 10.

Table 3 shows effects of dosimetric factors on grade ≥ 2 RP, adjusted for significant clinical factors in Table 2 (i.e. age, tumor diameter and distance from the bronchus). Six bronchial dosimetric factors were significantly correlated with grade ≥ 2 RP. Lung dosimetric factors were not significantly correlated with grade ≥ 2 RP. Among the significant bronchial dosimetric factors, bronchus V35_Gy_ had the highest hazard ratio (HR) (HR 1.24, 95% CI 1.03–1.49, *P* = 0.027). The results of the multivariate analysis, including bronchus V35_Gy_ and significant clinical factors of age, tumor diameter and distance from the bronchus, are presented in Table 4. Age (HR 3.78, 95% CI 1.07–13.35, *P* = 0.039), tumor diameter (HR 6.31, 95% CI 1.40–28.51, *P* = 0.017) and bronchus V35_Gy_ remained significant factors for grade ≥ 2 RP.

**Table 3:** Effects of dosimetric factors on grade ≥ 2 radiation pneumonitis after adjustment for clinical factors of *P*-value <0.05 (i.e. age, tumor diameter and distance from bronchus) in Table 2

**Factors**	**HR**	**95%CI**	***P*-value**
BED_10_	1.06	0.98–1.15	0.13
Bronchus Dmax (Gy)	1.04	0.96–1.12	0.38
Bronchus Dmean (Gy)	1.03	0.92–1.15	0.61
Bronchus D0.1_cc_ (Gy)	1.03	0.97–1.10	0.33
Bronchus D0.2_cc_ (Gy)	1.03	0.98–1.08	0.30
Bronchus D0.3_cc_ (Gy)	1.03	0.98–1.08	0.27
Bronchus D0.4_cc_ (Gy)	1.03	0.98–1.08	0.27
Bronchus D0.5_cc_ (Gy)	1.03	0.98–1.08	0.27
Bronchus D1_cc_ (Gy)	1.02	0.98–1.07	0.39
Bronchus D1.5_cc_ (Gy)	1.02	0.97–1.07	0.41
Bronchus D2_cc_ (Gy)	1.02	0.97–1.07	0.48
Bronchus D3_cc_ (Gy)	1.03	0.98–1.09	0.25
Bronchus D4_cc_ (Gy)	1.04	0.98–1.10	0.19
Bronchus D5_cc_ (Gy)	1.04	0.98–1.10	0.22
Bronchus V50_Gy_ (cc)	0.17	0.00–29.19	0.50
Bronchus V45_Gy_ (cc)	1.03	0.20–5.30	0.97
Bronchus V40_Gy_ (cc)	1.63	0.85–3.12	0.14
Bronchus V35_Gy_ (cc)	1.24	1.03–1.49	0.027
Bronchus V30_Gy_ (cc)	1.16	1.03–1.32	0.015
Bronchus V25_Gy_ (cc)	1.13	1.03–1.23	0.007
Bronchus V20_Gy_ (cc)	1.11	1.01–1.20	0.022
Bronchus V15_Gy_ (cc)	1.09	1.02–1.16	0.008
Bronchus V10_Gy_ (cc)	1.08	1.01–1.16	0.018
MLD (Gy)	0.91	0.64–1.28	0.57
Lung V20_Gy_ (%)	0.93	0.80–1.09	0.38
Lung V10_Gy_ (%)	0.99	0.89–1.10	0.82
Lung V8_Gy_ (%)	1.00	0.90–1.11	0.98
Lung V5_Gy_ (%)	0.98	0.91–1.07	0.70

HR, hazard ratio; 95% CI, 95% confidence interval; Dx cc, dose absorbed by the most exposed x cubic centimeters of the bronchus; Vn, volume of the bronchus or lung receiving a minimum dose of n Gy; MLD, mean lung dose.

**Table 4:** Multivariate analysis for grade ≥ 2 radiation pneumonitis

**Factors**	**HR**	**95% CI**	***P*-value**
Age (>77 vs. ≤77 years)	3.78	1.07–13.35	0.039
Tumor diameter (>26 vs. ≤26 mm)	6.31	1.40–28.51	0.017
Distance from the bronchus (≤1.9 vs. >1.9 cm)	1.43	0.45–4.53	0.54
Bronchus V35_Gy_ (cc)	1.24	1.03–1.49	0.027

HR, hazard ratio; 95% CI, 95% confidence interval; Vn, volume of the bronchus receiving a minimum dose of n Gy.

**Fig. 2:**
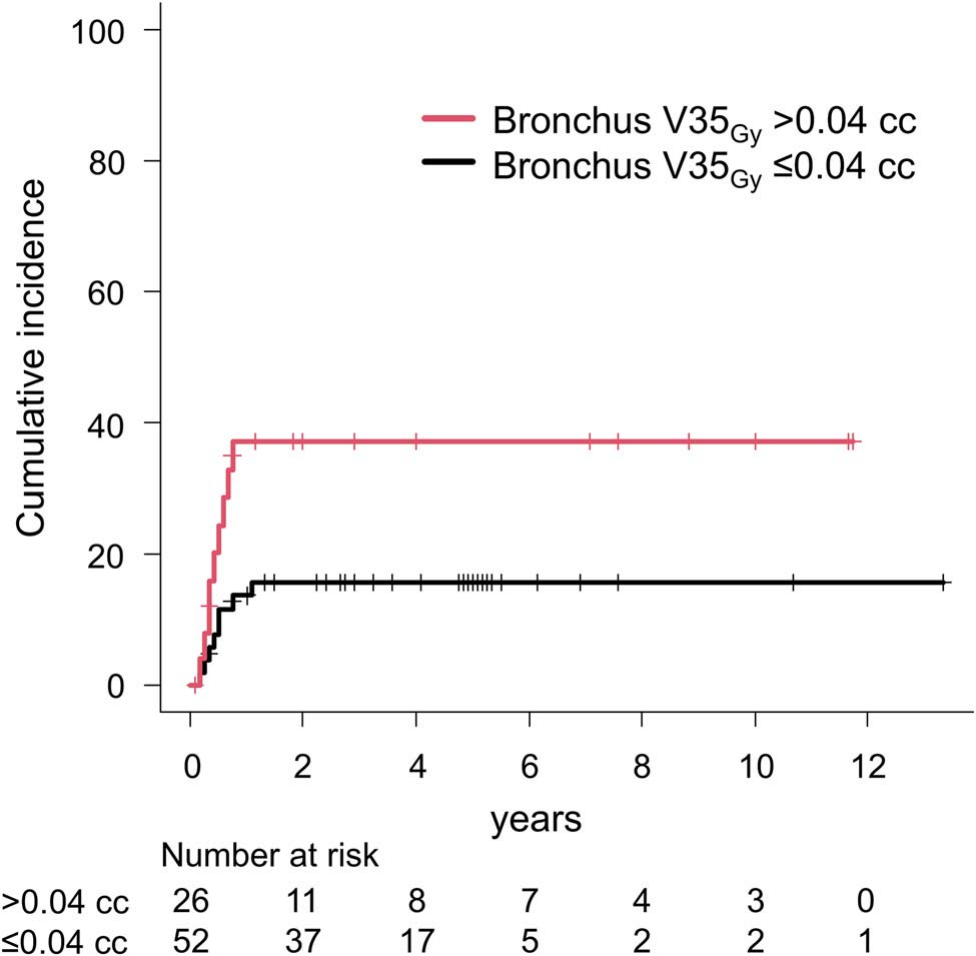
Differences in the cumulative incidence of grade ≥ 2 radiation pneumonitis based on the bronchus V35_Gy_ threshold.

The optimal threshold and area under the curve (AUC) for bronchus V35_Gy_ were determined using ROC curve analysis (AUC: 0.632, threshold: 0.04 cc). The 4-year cumulative incidence of grade ≥ 2 RP was compared between values above and below the ROC threshold using Gray’s test and Fig. 2 summarizes the results. The 4-year incidence of grade ≥ 2 RP in the bronchus V35_Gy_ ≤ 0.04 cc vs. >0.04 cc groups was 15.7% vs. 37.0% (*P* = 0.036).

The results of the Fine-Gray model and ROC curve analysis suggest that the bronchial dose is more crucial than the lung dose for preventing the onset of RP in SBRT for central tumors. Therefore, it is recommended to maintain bronchus V35_Gy_ < 0.04 cc as a dose constraint of grade ≥ 2 RP.

## DISCUSSION

We examined the bronchial dose in SBRT for central early-stage NSCLC and investigated risk factors for grade ≥ 2 RP. Among 78 patients, 17 and six developed grade ≥ 2 and ≥ 3 RP after SBRT, revealing that the 4-year cumulative incidence of grade ≥ 2 and ≥ 3 RP was 22.5% and 8.5%, respectively. The incidence of symptomatic RP was previously reported to range between 10 and 15% after SBRT [18, 26, 27]. However, when limited to the central tumor, severe toxicities, including treatment-related death, were frequently reported, even if risk-adapted dose fractionation regimens were attempted [15–17]. When 18 patients with tumors adjacent to the proximal bronchial tree received 45–50 Gy in five fractions, four treatment-related deaths (22%) were reported [15]. When limited to ‘ultra-central’ tumors, a subset of central tumors abutting the central airway [28], among 47 patients who received SBRT with 60 Gy over 12 fractions, grade ≥ 3 toxicity was observed in 18 patients (38%) and fatal pulmonary hemorrhage (15%) in seven [16]. A recent systematic review and meta-analysis of SBRT for ultra-central tumors reported a risk of grades 3–4 toxicity of 6%, which was predominantly related to RP. The pooled grade 5 toxicity risk was 4%, with most events (58%) being attributed to hemoptysis [29]. In the present study, which focused on the central tumor, the 4-year cumulative incidence of grade ≥ 2 and ≥ 3 RP was 22.5% and 8.5% and there was no grade 4 RP or treatment-related death. These rates are slightly high in SBRT, but low for central tumors. There are three possible reasons for this result. Eight patients were treated with risk-adapted dose fractionation regimens, mainly 60 Gy in eight fractions. The introduction of 60 Gy in eight fractions currently appears to have achieved consensus in SBRT for central or ultra-central tumors [29, 30]. Furthermore, although exact numbers were not known, some patients, particularly those with ultra-central tumors, may had received conventional radiotherapy instead of SBRT in our institution. Therefore, in the present study, fewer patients may have been at a high risk of severe toxicities. The European Society of Medical Oncology guidelines suggest more conventional radiotherapy for large tumors or central NSCLC instead of SBRT [14]. Moreover, the lower incidence of symptomatic RP may be attributed to the selection of tumors located within 3 cm of the trachea as the subject of analysis.

The reasons for central tumors being a risk factor may include inflammation of the bronchus, lymphatic flow and increased radiation doses to the lungs [19, 20]. Thus, it is important to clarify whether doses to the lung or bronchus will contribute more to the development of RP. In the multivariate analysis, six bronchial dosimetric factors were significantly associated with grade ≥ 2 RP, whereas lung dosimetric factors were not. This result suggests that the bronchial dose was more closely associated with the development of RP than the lung dose in central tumors. Regarding SBRT targeting central tumors, bronchial dose constraints may be more crucial than lung doses in preventing the development of RP. Specifically, bronchus V35_Gy_ < 0.04 cc is recommended for preventing grade ≥ 2 RP in SBRT for central early-stage NSCLC. Timmerman developed dose constraints using a universal model and concluded that large bronchus V42.4_Gy_ < 5 cc is recommended [31]. In our study, however, the constraints were derived from actual clinical data, which may lead to differences from those proposed by Timmerman. Patients with tumors within 3 cm of the main or lobar bronchus were included in the present study. Therefore, since fewer patients received high doses of radiation to the bronchi, the high dose area, such as V40–50_Gy_, may not have significantly affected the development of grade ≥ 2 RP.

In the multivariate analysis, age > 77 years and a tumor diameter > 26 mm remained significant risk factors for grade ≥ 2 RP among several clinical factors. Elderly patients are considered to have lower tolerance than younger patients, which may contribute to a higher incidence of grade ≥ 2 RP. Additionally, as shown in Supplementary Table 1, larger tumors result in higher radiation doses to the bronchus and lung, potentially leading to an increased incidence of grade ≥ 2 RP.

‘Central’ is now widened to include the region within 2 cm in all directions of any mediastinal critical structure, including the bronchial tree, esophagus and heart [13]. In the present study, the subject was defined as a tumor located within 3 cm of the bronchus. This definition was selected for two main reasons. In assessments of bronchial doses, there are various factors to consider when setting margins, such as tumor motion and tumor size. The location of the lobe and tumor diameter both correlated with the dose received by the bronchus, as shown in Supplementary Table 1. Furthermore, in this study, we evaluated not only high-dose regions, such as the maximum dose, but also moderate dose regions, including the mean dose and D1_cc_.

The present study had several limitations. First, this was a retrospective study conducted at a single institution. Therefore, it possessed the inherent biases typical of retrospective studies. To validate the results obtained, data from a large-scale prospective study are necessary. Second, in this study, prescriptions were made to the isocenter. In SBRT for early-stage NSCLC, prescribing to D95% of PTV is now widely adopted [8]. Our institution follows this practice for SBRT for early-stage NSCLC, with plans to analyze these data in the future. Third, we acknowledge the presence of hemoptysis as a potential adverse event associated with the bronchial tract. However, since we encountered only one case of hemoptysis in the present study, our research focused exclusively on RP. Fourth, the present study included patients with a low risk of severe adverse events in SBRT for central tumors because not only high-dose parameters, such as the maximum dose, but also moderate-dose parameters needed to be evaluated. Fifth, in this study, cases treated with six or eight fractions were converted to an equivalent four-fraction schedule using the LQ model. Since the LQ model is known to deviate from actual measurements in high-dose regions, caution is required when interpreting the results.

In conclusion, we investigated risk factors and bronchial dose constraints for symptomatic RP in SBRT for central early-stage NSCLC. Among 78 patients received SBRT in a median prescribed dose of 50 Gy in four fractions, the 4-year cumulative incidence of grades ≥ 2 and ≥ 3 RP was 22.5% and 8.5%, respectively. The present study demonstrated that age and the tumor diameter were independent risk factors associated with the development of grade ≥ 2 RP. When SBRT is employed for central tumors, the doses of the bronchus may contribute more to the development of RP than lung doses. In SBRT for central early-stage NSCLC, bronchus V35_Gy_ < 0.04 cc may be the most effective indicator among dose parameters for preventing grade ≥ 2 RP.

## Supplementary Material

Supplementary_Table_1_rraf016

## PRESENTATION AT A CONFERENCE

This study was presented at the 66th ASTRO annual meeting.
