# Supplementary material for: Stereotactic body radiotherapy for central non-small cell lung cancer: risk analysis of radiation pneumonitis and bronchial dose constraints
Source: J Radiat Res. 2025 Apr 14;66(3):264–71. doi: 10.1093/jrr/rraf016 (PMC12100488; doi:10.1093/jrr/rraf016)
Supplement: Supplementary_Table_1_rraf016 [file supplementary_table_1_rraf016.docx]

**Supplementary Table 1. Mean values of bronchial parameters according to each clinical factor**

| **Characteristics** | **Number** |  | **Mean dose (Gy)** | | |  | **Max dose (Gy)** | | |  | | **Bronchus D1_cc_ (Gy)** | | | |
| --- | --- | --- | --- | --- | --- | --- | --- | --- | --- | --- | --- | --- | --- | --- | --- |
|  |  |  | **Mean** | **Range** | ***p*-value** |  | **Mean** | **Range** | ***p*-value** |  | **Mean** | | **Range** | ***p*-value** |  |
| Age (years) |  |  |  |  | 0.72 |  |  |  | 0.11 |  |  | |  | 0.14 |  |
| ≤77 | 41 |  | 5.39 | 0.47–19.81 |  |  | 29.58 | 4.42–53.47 |  |  | 15.35 | | 1.25–43.87 |  |  |
| >77 | 37 |  | 5.75 | 0.46–20.58 |  |  | 34.93 | 1.97–54.68 |  |  | 19.80 | | 0.94–48.86 |  |  |
| Sex |  |  |  |  | 0.60 |  |  |  | 0.59 |  |  | |  | 0.92 |  |
| Male | 43 |  | 5.32 | 0.46–20.58 |  |  | 31.30 | 1.97–53.47 |  |  | 17.60 | | 0.94–43.87 |  |  |
| Female | 35 |  | 5.86 | 0.85–19.81 |  |  | 33.12 | 6.02–54.68 |  |  | 17.29 | | 1.69–48.86 |  |  |
| PS |  |  |  |  | 0.95 |  |  |  | 0.17 |  |  | |  | 0.29 |  |
| 0, 1 | 71 |  | 5.55 | 0.46–20.58 |  |  | 31.38 | 1.97–54.68 |  |  | 16.96 | | 0.94–44.58 |  |  |
| 2 | 7 |  | 5.67 | 1.04–13.02 |  |  | 39.56 | 15.50–50.72 |  |  | 22.55 | | 1.42–48.86 |  |  |
| FEV_1_ (L) |  |  |  |  | 0.44 |  |  |  | 0.80 |  |  | |  | 0.90 |  |
| ≤1.49 | 39 |  | 5.17 | 0.46–19.81 |  |  | 32.55 | 1.97–53.47 |  |  | 17.65 | | 0.94–48.86 |  |  |
| >1.49 | 39 |  | 5.95 | 0.47–20.58 |  |  | 31.68 | 4.42–54.68 |  |  | 17.27 | | 1.25–44.58 |  |  |
| Tumor location |  |  |  |  | 0.13 |  |  |  | 0.024 |  |  | |  | 0.025 |  |
| Upper or middle lobe | 50 |  | 4.98 | 0.46–19.81 |  |  | 29.30 | 1.97–50.86 |  |  | 14.97 | | 0.94–48.86 |  |  |
| Lower lobe | 28 |  | 6.59 | 1.04–20.58 |  |  | 37.15 | 15.19–54.68 |  |  | 21.90 | | 1.42–44.58 |  |  |
| Tumor diameter (mm) |  |  |  |  | 0.006 |  |  |  | 0.01 |  |  | |  | 0.014 |  |
| ≤26 | 41 |  | 4.26 | 0.47–10.97 |  |  | 28.03 | 4.42–54.68 |  |  | 14.02 | | 1.25–44.58 |  |  |
| >26 | 37 |  | 7.00 | 0.46–20.58 |  |  | 36.64 | 1.97–53.47 |  |  | 21.28 | | 0.94–48.86 |  |  |
| Distance from the bronchus (cm) |  |  |  |  | <0.001 |  |  |  | <0.001 |  |  | |  | <0.001 |  |
| ≤1 | 23 |  | 9.49 | 1.88–20.58 |  |  | 47.80 | 32.45–54.68 |  |  | 32.25 | | 11.50–48.86 |  |  |
| 1–2 | 19 |  | 4.66 | 0.67–10.84 |  |  | 36.65 | 9.67–50.08 |  |  | 14.75 | | 1.42–33.76 |  |  |
| 2–3 | 36 |  | 3.53 | 0.46–13.80 |  |  | 19.70 | 1.97–36.20 |  |  | 9.44 | | 0.94–24.85 |  |  |

Dx cc, dose absorbed by the most exposed x cubic centimeters of the bronchus; PS, performance status; FEV_1_, forced expiratory volume in 1 second.
